# 1-Diphenyl­phosphino-1′-(diphenyl­phosphinoyl)cobaltocenium hexa­fluorido­phosphate

**DOI:** 10.1107/S1600536808027992

**Published:** 2008-09-06

**Authors:** Xiang-Hua Wu, Rui Guo, Shan Jin, Guang-Ao Yu, Sheng-Hua Liu

**Affiliations:** a Key Laboratory of Pesticides and Chemical Biology, College of Chemistry, Central China Normal University, Wuhan 430079, People’s Republic of China

## Abstract

The title compound, [Co(C_17_H_14_OP)(C_17_H_14_P)]PF_6_, was obtained unintentionally as the product of an attempted synthesis of [1,1′-bis­(oxodiphenyl­phospho­ranyl)cobaltocenium] hexa­fluorido­phosphate. The O atom of the oxo group is disordered over two positions with site occupancies of 0.65:0.35. The crystal structure contains weak inter­molecular C—H⋯F hydrogen bonds, connecting the components of the structure into chains parallel to [010].

## Related literature

For related literature, see: Song (2004 ▶).
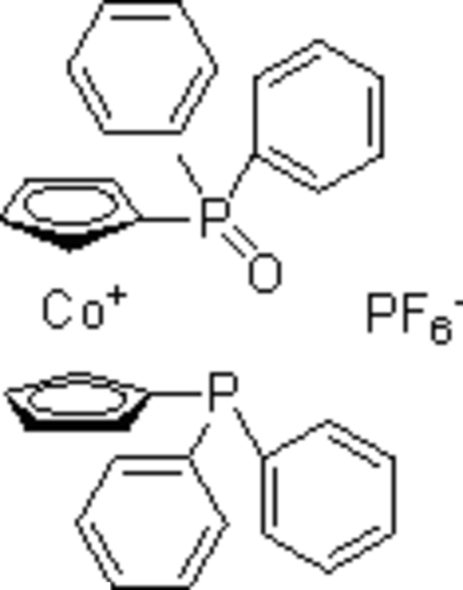


## Experimental

### 

#### Crystal data

[Co(C_17_H_14_OP)(C_17_H_14_P)]PF_6_
*M* *_r_* = 718.40
Monoclinic, 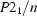

*a* = 12.0364 (14) Å
*b* = 10.7014 (13) Å
*c* = 24.973 (3) Å
β = 103.94°
*V* = 3121.9 (6) Å^3^
*Z* = 4
Mo *K*α radiation
μ = 0.77 mm^−1^
*T* = 293 (2) K
0.30 × 0.20 × 0.10 mm

#### Data collection

Bruker SMART CCD diffractometer
Absorption correction: none
33887 measured reflections
7444 independent reflections
3990 reflections with *I* > 2σ(*I*)
*R*_int_ = 0.093

#### Refinement

*R*[*F* ^2^ > 2σ(*F* ^2^)] = 0.053
*wR*(*F* ^2^) = 0.101
*S* = 0.85
7444 reflections
416 parameters
H-atom parameters constrained
Δρ_max_ = 0.40 e Å^−3^
Δρ_min_ = −0.50 e Å^−3^

### 

Data collection: *SMART* (Bruker, 2001 ▶); cell refinement: *SAINT* (Bruker, 2001 ▶); data reduction: *SAINT*; program(s) used to solve structure: *SHELXS97* (Sheldrick, 2008 ▶); program(s) used to refine structure: *SHELXL97* (Sheldrick, 2008 ▶); molecular graphics: *SHELXTL* (Sheldrick, 2008 ▶); software used to prepare material for publication: *SHELXTL*.

## Supplementary Material

Crystal structure: contains datablocks I, global. DOI: 10.1107/S1600536808027992/lh2674sup1.cif

Structure factors: contains datablocks I. DOI: 10.1107/S1600536808027992/lh2674Isup2.hkl

Additional supplementary materials:  crystallographic information; 3D view; checkCIF report

## Figures and Tables

**Table 1:** Hydrogen-bond geometry (Å, °)

*D*—H⋯*A*	*D*—H	H⋯*A*	*D*⋯*A*	*D*—H⋯*A*
C4—H4⋯F6^i^	0.98	2.49	3.450 (4)	167
C18—H18⋯F5	0.98	2.43	3.327 (4)	152

Symmetry code: (i) 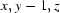.

**Fig. 1.**
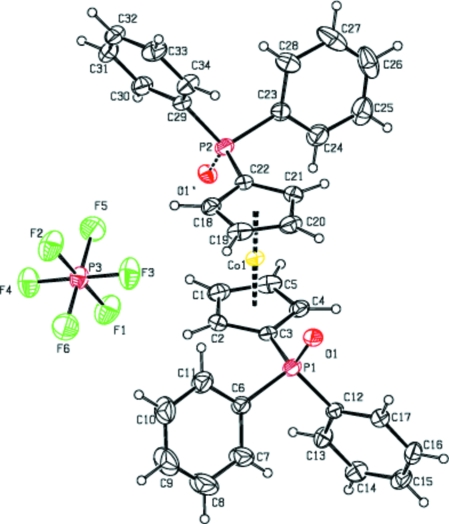
The molecular structure of (I), with atom labels and 50% probability displacement ellipsoids for non-H atoms. The minor component of disorder is bonded to P2.

**Fig. 2.**
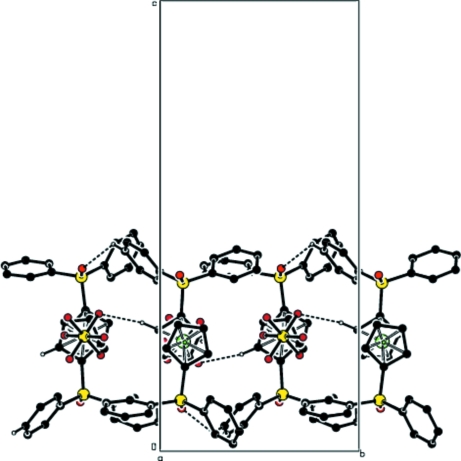
The packing of (I), viewed approximately along the c axis, showing molecules connected by C—H···F hydrogen bonds (dashed lines).

## supplementary crystallographic information

### Comment

The molecular structure of the title compound consists of a [(η5-Ph_2_POC_5_H_4_)(η5-Ph_2_PC_5_H_4_)Co]^+^ cation and a PF6^-^ anion, (I) (Fig. 1), which essentially identical to the isomorphous complex [(η5Ph_2_POC_5_H_4_)_2_Co]^+^(PF6)^-^ (II) (Song, 2004). The two substituted Cp rings are staggered and essentially parallel with a dihedral angle of 0.2 (3)° for (II) and 0.2 (9)° for (I). The distance between the Co atom and the centroid of Cp ring is 1.635 (9) Å for (II) and 1.644 (1) Å for (I). In both structures, the two Ph_2_P substituents are *trans* to each other with respect to the Co metal center. However, both P atoms are double bonded to two O atoms in (II) while in (I) the single O atom of the oxo group is disordered with an approximate ratio of occupancies of 0.65:0.35. The crystal structure contains weak intermolecular C—H···F hydrogen bonds, connecting the components of the structure into one-dimensional chains.

### Experimental

The title compound was obtained unintentionally as a side-product of synthesis of [1,1'-bis(oxodiphenylphosphoranyl) cobaltocenium] hexafluorophosphate (Song, 2004). Crystals appropriate for data collection were obtained by slow evaporation from dichloromethane and hexane solution at romm temperature.

### Refinement

All H atoms were placed in geometrically idealized positions and constrained to ride on their parent atoms with C—H = 0.93 Å; with *U*_iso_(H) = 1.2*U*_eq_(C).

### Crystal data

**Table:**

[Co(C_17_H_14_OP)(C_17_H_14_P)]PF_6_	*F*(000) = 1464
*M**_r_* = 718.40	*D*_x_ = 1.528 Mg m^−^^3^
Monoclinic, *P*2_1_/*n*	Mo *K*α radiation, λ = 0.71073 Å
Hall symbol: -P 2yn	Cell parameters from 3441 reflections
*a* = 12.0364 (14) Å	θ = 2.5–23.0°
*b* = 10.7014 (13) Å	µ = 0.77 mm^−^^1^
*c* = 24.973 (3) Å	*T* = 293 K
β = 103.94°	Block, yellow
*V* = 3121.9 (6) Å^3^	0.30 × 0.20 × 0.10 mm
*Z* = 4	

### Data collection

**Table:**

Bruker SMART CCD diffractometer	3990 reflections with *I* > 2σ(*I*)
Radiation source: fine-focus sealed tube	*R*_int_ = 0.093
graphite	θ_max_ = 28.0°, θ_min_ = 1.7°
φ and ω scans	*h* = −15→15
33887 measured reflections	*k* = −13→14
7444 independent reflections	*l* = −32→32

### Refinement

**Table:**

Refinement on *F*^2^	Primary atom site location: structure-invariant direct methods
Least-squares matrix: full	Secondary atom site location: difference Fourier map
*R*[*F*^2^ > 2σ(*F*^2^)] = 0.053	Hydrogen site location: inferred from neighbouring sites
*wR*(*F*^2^) = 0.101	H-atom parameters constrained
*S* = 0.85	*w* = 1/[σ^2^(*F*_o_^2^) + (0.0333*P*)^2^] where *P* = (*F*_o_^2^ + 2*F*_c_^2^)/3
7444 reflections	(Δ/σ)_max_ < 0.001
416 parameters	Δρ_max_ = 0.40 e Å^−^^3^
0 restraints	Δρ_min_ = −0.50 e Å^−^^3^

### Special details

**Table:**

Geometry. All e.s.d.'s (except the e.s.d. in the dihedral angle between two l.s. planes) are estimated using the full covariance matrix. The cell e.s.d.'s are taken into account individually in the estimation of e.s.d.'s in distances, angles and torsion angles; correlations between e.s.d.'s in cell parameters are only used when they are defined by crystal symmetry. An approximate (isotropic) treatment of cell e.s.d.'s is used for estimating e.s.d.'s involving l.s. planes.
Refinement. Refinement of *F*^2^ against ALL reflections. The weighted *R*-factor *wR* and goodness of fit *S* are based on *F*^2^, conventional *R*-factors *R* are based on *F*, with *F* set to zero for negative *F*^2^. The threshold expression of *F*^2^ > σ(*F*^2^) is used only for calculating *R*-factors(gt) *etc*. and is not relevant to the choice of reflections for refinement. *R*-factors based on *F*^2^ are statistically about twice as large as those based on *F*, and *R*- factors based on ALL data will be even larger.

### Fractional atomic coordinates and isotropic or equivalent isotropic displacement parameters (Å^2^)

**Table:**

	*x*	*y*	*z*	*U*_iso_*/*U*_eq_	Occ. (<1)
Co1	0.48773 (3)	0.11791 (3)	0.243490 (14)	0.03887 (12)	
P1	0.44687 (7)	0.11498 (7)	0.37385 (3)	0.0426 (2)	
P2	0.53221 (7)	0.10378 (7)	0.11305 (3)	0.0424 (2)	
O1	0.5650 (2)	0.0989 (2)	0.39072 (11)	0.0433 (9)	0.648 (3)
O1'	0.4184 (4)	0.1082 (5)	0.0948 (2)	0.0420 (16)	0.352 (3)
C1	0.3191 (2)	0.1470 (3)	0.20725 (12)	0.0539 (8)	
H1	0.2886	0.1911	0.1724	0.065*	
C2	0.3483 (2)	0.2018 (3)	0.26038 (11)	0.0457 (7)	
H2	0.3416	0.2906	0.2687	0.055*	
C3	0.3895 (2)	0.1058 (2)	0.29970 (10)	0.0384 (7)	
C4	0.3851 (2)	−0.0078 (3)	0.26934 (12)	0.0464 (8)	
H4	0.4083	−0.0905	0.2850	0.056*	
C5	0.3415 (3)	0.0194 (3)	0.21322 (12)	0.0555 (9)	
H5	0.3298	−0.0413	0.1830	0.067*	
C6	0.3991 (3)	0.2628 (3)	0.39517 (11)	0.0426 (7)	
C7	0.3226 (3)	0.2705 (3)	0.42831 (13)	0.0692 (10)	
H7	0.2885	0.1982	0.4375	0.083*	
C8	0.2965 (4)	0.3844 (4)	0.44781 (16)	0.0926 (13)	
H8	0.2441	0.3887	0.4697	0.111*	
C9	0.3464 (4)	0.4900 (4)	0.43534 (16)	0.0918 (14)	
H9	0.3290	0.5664	0.4491	0.110*	
C10	0.4226 (3)	0.4851 (3)	0.40253 (15)	0.0773 (11)	
H10	0.4565	0.5580	0.3938	0.093*	
C11	0.4489 (3)	0.3710 (3)	0.38234 (12)	0.0608 (9)	
H11	0.5005	0.3676	0.3600	0.073*	
C12	0.3648 (2)	−0.0022 (2)	0.39898 (10)	0.0362 (7)	
C13	0.2468 (3)	−0.0150 (3)	0.37922 (11)	0.0478 (8)	
H13	0.2079	0.0355	0.3505	0.057*	
C14	0.1879 (3)	−0.1019 (3)	0.40198 (12)	0.0527 (8)	
H14	0.1091	−0.1099	0.3889	0.063*	
C15	0.2459 (3)	−0.1766 (3)	0.44402 (12)	0.0523 (8)	
H15	0.2059	−0.2355	0.4594	0.063*	
C16	0.3609 (3)	−0.1659 (3)	0.46360 (12)	0.0526 (8)	
H16	0.3990	−0.2169	0.4923	0.063*	
C17	0.4219 (2)	−0.0788 (2)	0.44078 (11)	0.0430 (7)	
H17	0.5009	−0.0724	0.4537	0.052*	
C18	0.5894 (2)	0.2405 (3)	0.21579 (11)	0.0466 (8)	
H18	0.5657	0.3218	0.1988	0.056*	
C19	0.6330 (3)	0.2183 (3)	0.27246 (12)	0.0550 (9)	
H19	0.6442	0.2816	0.3016	0.066*	
C20	0.6561 (2)	0.0906 (3)	0.28049 (12)	0.0529 (8)	
H20	0.6865	0.0492	0.3160	0.064*	
C21	0.6280 (2)	0.0324 (3)	0.22828 (11)	0.0459 (7)	
H21	0.6349	−0.0571	0.2214	0.055*	
C22	0.5869 (2)	0.1245 (2)	0.18724 (10)	0.0376 (7)	
C23	0.5941 (3)	−0.0459 (3)	0.10227 (10)	0.0433 (7)	
C24	0.5394 (3)	−0.1523 (3)	0.11334 (12)	0.0645 (10)	
H24	0.4721	−0.1451	0.1252	0.077*	
C25	0.5828 (4)	−0.2700 (3)	0.10713 (13)	0.0790 (12)	
H25	0.5465	−0.3412	0.1158	0.095*	
C26	0.6799 (4)	−0.2798 (4)	0.08800 (15)	0.0883 (15)	
H26	0.7091	−0.3583	0.0831	0.106*	
C27	0.7333 (3)	−0.1766 (4)	0.07628 (15)	0.0860 (13)	
H27	0.7988	−0.1847	0.0630	0.103*	
C28	0.6925 (3)	−0.0587 (3)	0.08360 (12)	0.0617 (9)	
H28	0.7312	0.0117	0.0760	0.074*	
C29	0.6164 (2)	0.2193 (2)	0.08749 (10)	0.0382 (7)	
C30	0.5673 (3)	0.2768 (3)	0.03768 (11)	0.0519 (8)	
H30	0.4934	0.2555	0.0187	0.062*	
C31	0.6278 (3)	0.3662 (3)	0.01609 (13)	0.0616 (9)	
H31	0.5944	0.4045	−0.0173	0.074*	
C32	0.7358 (3)	0.3977 (3)	0.04378 (14)	0.0586 (9)	
H32	0.7759	0.4579	0.0292	0.070*	
C33	0.7860 (3)	0.3415 (3)	0.09278 (13)	0.0611 (9)	
H33	0.8600	0.3632	0.1115	0.073*	
C34	0.7262 (3)	0.2522 (3)	0.11448 (12)	0.0528 (8)	
H34	0.7607	0.2139	0.1478	0.063*	
P3	0.50693 (7)	0.61873 (8)	0.25491 (3)	0.0511 (2)	
F1	0.41059 (16)	0.51900 (17)	0.25695 (8)	0.0855 (6)	
F2	0.60294 (16)	0.71804 (17)	0.25270 (8)	0.0873 (6)	
F3	0.59715 (17)	0.53606 (17)	0.29675 (8)	0.0860 (6)	
F4	0.41560 (16)	0.70120 (16)	0.21294 (7)	0.0771 (6)	
F5	0.53356 (19)	0.54514 (18)	0.20439 (8)	0.0898 (7)	
F6	0.47884 (19)	0.69185 (18)	0.30484 (8)	0.0942 (7)	

### Atomic displacement parameters (Å^2^)

**Table:**

	*U*^11^	*U*^22^	*U*^33^	*U*^12^	*U*^13^	*U*^23^
Co1	0.0426 (3)	0.0416 (2)	0.0368 (2)	0.00034 (19)	0.01813 (18)	0.00186 (18)
P1	0.0536 (5)	0.0389 (5)	0.0401 (4)	−0.0040 (4)	0.0208 (4)	0.0019 (4)
P2	0.0501 (5)	0.0414 (5)	0.0404 (4)	−0.0045 (4)	0.0199 (4)	0.0011 (4)
O1	0.0373 (19)	0.0472 (19)	0.0474 (18)	0.0000 (15)	0.0142 (14)	0.0019 (14)
O1'	0.023 (3)	0.052 (4)	0.048 (3)	0.007 (3)	0.003 (2)	0.003 (3)
C1	0.042 (2)	0.079 (3)	0.0432 (19)	0.0051 (17)	0.0157 (15)	0.0124 (17)
C2	0.0436 (19)	0.0467 (19)	0.0527 (19)	0.0094 (15)	0.0227 (15)	0.0052 (15)
C3	0.0396 (17)	0.0414 (17)	0.0408 (16)	−0.0008 (14)	0.0223 (13)	0.0024 (13)
C4	0.057 (2)	0.0397 (18)	0.0495 (19)	−0.0095 (15)	0.0270 (16)	−0.0022 (14)
C5	0.052 (2)	0.071 (2)	0.047 (2)	−0.0192 (18)	0.0208 (16)	−0.0151 (17)
C6	0.053 (2)	0.0382 (17)	0.0391 (17)	−0.0036 (15)	0.0167 (15)	−0.0039 (13)
C7	0.086 (3)	0.054 (2)	0.081 (2)	−0.009 (2)	0.048 (2)	−0.0186 (19)
C8	0.111 (4)	0.073 (3)	0.114 (3)	0.001 (3)	0.066 (3)	−0.030 (3)
C9	0.125 (4)	0.057 (3)	0.096 (3)	0.014 (3)	0.031 (3)	−0.027 (2)
C10	0.101 (3)	0.042 (2)	0.089 (3)	−0.002 (2)	0.024 (2)	−0.002 (2)
C11	0.078 (3)	0.049 (2)	0.062 (2)	−0.0080 (19)	0.0274 (19)	0.0015 (17)
C12	0.0411 (18)	0.0366 (16)	0.0340 (15)	−0.0025 (13)	0.0153 (13)	0.0003 (12)
C13	0.050 (2)	0.0490 (19)	0.0421 (17)	−0.0015 (16)	0.0055 (15)	0.0072 (14)
C14	0.0417 (19)	0.061 (2)	0.055 (2)	−0.0108 (17)	0.0094 (16)	−0.0007 (17)
C15	0.064 (2)	0.0449 (19)	0.053 (2)	−0.0172 (17)	0.0225 (18)	−0.0004 (15)
C16	0.069 (2)	0.0422 (18)	0.0461 (19)	0.0018 (17)	0.0121 (17)	0.0097 (14)
C17	0.0431 (18)	0.0427 (17)	0.0447 (17)	−0.0002 (14)	0.0133 (15)	0.0021 (14)
C18	0.053 (2)	0.0413 (18)	0.0521 (19)	−0.0065 (15)	0.0251 (16)	0.0007 (15)
C19	0.052 (2)	0.070 (2)	0.048 (2)	−0.0159 (18)	0.0217 (16)	−0.0145 (17)
C20	0.044 (2)	0.076 (2)	0.0401 (18)	0.0032 (18)	0.0132 (15)	0.0096 (16)
C21	0.0423 (19)	0.0506 (19)	0.0490 (18)	0.0083 (15)	0.0192 (15)	0.0037 (15)
C22	0.0383 (17)	0.0407 (17)	0.0388 (15)	0.0007 (14)	0.0193 (13)	0.0031 (13)
C23	0.052 (2)	0.0447 (18)	0.0339 (16)	0.0005 (15)	0.0114 (14)	−0.0037 (13)
C24	0.094 (3)	0.050 (2)	0.057 (2)	−0.006 (2)	0.032 (2)	−0.0042 (17)
C25	0.134 (4)	0.050 (2)	0.052 (2)	−0.001 (2)	0.018 (2)	−0.0026 (18)
C26	0.132 (4)	0.065 (3)	0.052 (2)	0.044 (3)	−0.009 (3)	−0.015 (2)
C27	0.073 (3)	0.109 (3)	0.074 (3)	0.034 (3)	0.012 (2)	−0.037 (3)
C28	0.062 (2)	0.068 (2)	0.058 (2)	0.0043 (19)	0.0204 (18)	−0.0164 (18)
C29	0.0410 (18)	0.0418 (17)	0.0359 (16)	0.0007 (14)	0.0174 (14)	0.0029 (13)
C30	0.044 (2)	0.065 (2)	0.0478 (18)	0.0021 (17)	0.0143 (15)	0.0093 (16)
C31	0.066 (2)	0.063 (2)	0.060 (2)	0.0059 (19)	0.0253 (19)	0.0265 (18)
C32	0.074 (3)	0.047 (2)	0.066 (2)	−0.0040 (19)	0.039 (2)	0.0085 (17)
C33	0.057 (2)	0.066 (2)	0.064 (2)	−0.0230 (18)	0.0229 (18)	−0.0048 (18)
C34	0.051 (2)	0.064 (2)	0.0431 (18)	−0.0105 (17)	0.0095 (16)	0.0072 (15)
P3	0.0522 (5)	0.0421 (5)	0.0566 (5)	−0.0059 (4)	0.0085 (4)	0.0039 (4)
F1	0.0773 (15)	0.0751 (14)	0.1006 (15)	−0.0315 (12)	0.0146 (12)	0.0130 (12)
F2	0.0744 (14)	0.0726 (14)	0.1087 (16)	−0.0314 (11)	0.0100 (12)	0.0197 (12)
F3	0.0777 (15)	0.0690 (13)	0.0979 (15)	0.0065 (11)	−0.0053 (12)	0.0259 (11)
F4	0.0703 (14)	0.0670 (13)	0.0819 (13)	0.0063 (11)	−0.0052 (11)	0.0120 (10)
F5	0.1254 (19)	0.0698 (14)	0.0855 (14)	0.0066 (13)	0.0477 (13)	−0.0058 (11)
F6	0.138 (2)	0.0778 (15)	0.0731 (14)	0.0089 (14)	0.0385 (13)	−0.0093 (11)

### Geometric parameters (Å, °)

**Table:**

Co1—C18	2.026 (3)	C14—H14	0.9300
Co1—C19	2.031 (3)	C15—C16	1.358 (4)
Co1—C4	2.034 (3)	C15—H15	0.9300
Co1—C5	2.035 (3)	C16—C17	1.391 (4)
Co1—C21	2.035 (3)	C16—H16	0.9300
Co1—C20	2.035 (3)	C17—H17	0.9300
Co1—C2	2.035 (3)	C18—C19	1.406 (4)
Co1—C1	2.037 (3)	C18—C22	1.428 (3)
Co1—C3	2.046 (2)	C18—H18	0.9800
Co1—C22	2.052 (2)	C19—C20	1.398 (4)
P1—O1	1.393 (3)	C19—H19	0.9800
P1—C12	1.800 (3)	C20—C21	1.411 (4)
P1—C6	1.807 (3)	C20—H20	0.9800
P1—C3	1.816 (3)	C21—C22	1.422 (3)
P2—O1'	1.337 (5)	C21—H21	0.9800
P2—C29	1.810 (3)	C23—C24	1.376 (4)
P2—C23	1.814 (3)	C23—C28	1.380 (4)
P2—C22	1.824 (3)	C24—C25	1.387 (4)
C1—C5	1.392 (4)	C24—H24	0.9300
C1—C2	1.415 (4)	C25—C26	1.368 (5)
C1—H1	0.9800	C25—H25	0.9300
C2—C3	1.425 (3)	C26—C27	1.345 (5)
C2—H2	0.9800	C26—H26	0.9300
C3—C4	1.428 (3)	C27—C28	1.382 (4)
C4—C5	1.404 (4)	C27—H27	0.9300
C4—H4	0.9800	C28—H28	0.9300
C5—H5	0.9800	C29—C34	1.377 (4)
C6—C11	1.377 (4)	C29—C30	1.386 (3)
C6—C7	1.381 (4)	C30—C31	1.387 (4)
C7—C8	1.377 (4)	C30—H30	0.9300
C7—H7	0.9300	C31—C32	1.360 (4)
C8—C9	1.350 (5)	C31—H31	0.9300
C8—H8	0.9300	C32—C33	1.367 (4)
C9—C10	1.370 (5)	C32—H32	0.9300
C9—H9	0.9300	C33—C34	1.383 (4)
C10—C11	1.386 (4)	C33—H33	0.9300
C10—H10	0.9300	C34—H34	0.9300
C11—H11	0.9300	P3—F6	1.5764 (19)
C12—C17	1.374 (3)	P3—F2	1.5807 (19)
C12—C13	1.393 (3)	P3—F3	1.5834 (18)
C13—C14	1.373 (4)	P3—F5	1.584 (2)
C13—H13	0.9300	P3—F1	1.5858 (19)
C14—C15	1.370 (4)	P3—F4	1.5908 (18)
			
C18—Co1—C19	40.55 (10)	C6—C11—C10	120.4 (3)
C18—Co1—C4	178.43 (11)	C6—C11—H11	119.8
C19—Co1—C4	140.65 (12)	C10—C11—H11	119.8
C18—Co1—C5	138.43 (12)	C17—C12—C13	119.4 (3)
C19—Co1—C5	178.94 (13)	C17—C12—P1	117.7 (2)
C4—Co1—C5	40.37 (11)	C13—C12—P1	122.9 (2)
C18—Co1—C21	68.32 (12)	C14—C13—C12	120.3 (3)
C19—Co1—C21	67.80 (12)	C14—C13—H13	119.9
C4—Co1—C21	110.88 (12)	C12—C13—H13	119.9
C5—Co1—C21	112.35 (13)	C15—C14—C13	119.6 (3)
C18—Co1—C20	68.32 (12)	C15—C14—H14	120.2
C19—Co1—C20	40.23 (11)	C13—C14—H14	120.2
C4—Co1—C20	112.04 (12)	C16—C15—C14	120.9 (3)
C5—Co1—C20	140.53 (14)	C16—C15—H15	119.5
C21—Co1—C20	40.56 (11)	C14—C15—H15	119.5
C18—Co1—C2	112.42 (12)	C15—C16—C17	120.2 (3)
C19—Co1—C2	112.04 (12)	C15—C16—H16	119.9
C4—Co1—C2	68.41 (11)	C17—C16—H16	119.9
C5—Co1—C2	67.83 (12)	C12—C17—C16	119.6 (3)
C21—Co1—C2	178.76 (11)	C12—C17—H17	120.2
C20—Co1—C2	138.56 (12)	C16—C17—H17	120.2
C18—Co1—C1	111.63 (12)	C19—C18—C22	108.3 (3)
C19—Co1—C1	139.25 (14)	C19—C18—Co1	69.92 (16)
C4—Co1—C1	68.03 (12)	C22—C18—Co1	70.51 (15)
C5—Co1—C1	39.99 (11)	C19—C18—H18	125.9
C21—Co1—C1	140.21 (12)	C22—C18—H18	125.9
C20—Co1—C1	179.21 (13)	Co1—C18—H18	125.9
C2—Co1—C1	40.67 (10)	C20—C19—C18	108.8 (3)
C18—Co1—C3	140.52 (11)	C20—C19—Co1	70.04 (17)
C19—Co1—C3	112.30 (11)	C18—C19—Co1	69.53 (16)
C4—Co1—C3	40.96 (10)	C20—C19—H19	125.6
C5—Co1—C3	68.34 (11)	C18—C19—H19	125.6
C21—Co1—C3	137.93 (11)	Co1—C19—H19	125.6
C20—Co1—C3	110.86 (11)	C19—C20—C21	107.7 (3)
C2—Co1—C3	40.88 (10)	C19—C20—Co1	69.73 (18)
C1—Co1—C3	68.65 (11)	C21—C20—Co1	69.71 (16)
C18—Co1—C22	40.97 (10)	C19—C20—H20	126.2
C19—Co1—C22	68.44 (11)	C21—C20—H20	126.2
C4—Co1—C22	137.56 (11)	Co1—C20—H20	126.2
C5—Co1—C22	110.95 (11)	C20—C21—C22	108.9 (3)
C21—Co1—C22	40.73 (10)	C20—C21—Co1	69.73 (16)
C20—Co1—C22	68.66 (11)	C22—C21—Co1	70.30 (15)
C2—Co1—C22	140.47 (11)	C20—C21—H21	125.5
C1—Co1—C22	111.85 (11)	C22—C21—H21	125.5
C3—Co1—C22	178.34 (11)	Co1—C21—H21	125.5
O1—P1—C12	114.48 (15)	C21—C22—C18	106.3 (2)
O1—P1—C6	113.12 (15)	C21—C22—P2	128.7 (2)
C12—P1—C6	105.50 (13)	C18—C22—P2	124.9 (2)
O1—P1—C3	114.28 (14)	C21—C22—Co1	68.98 (15)
C12—P1—C3	102.26 (12)	C18—C22—Co1	68.52 (15)
C6—P1—C3	106.14 (12)	P2—C22—Co1	124.32 (14)
O1'—P2—C29	118.1 (2)	C24—C23—C28	118.5 (3)
O1'—P2—C23	113.9 (2)	C24—C23—P2	117.8 (2)
C29—P2—C23	105.42 (13)	C28—C23—P2	123.7 (2)
O1'—P2—C22	115.5 (2)	C23—C24—C25	121.1 (3)
C29—P2—C22	100.33 (12)	C23—C24—H24	119.4
C23—P2—C22	101.40 (12)	C25—C24—H24	119.4
C5—C1—C2	108.0 (3)	C26—C25—C24	119.1 (4)
C5—C1—Co1	69.90 (18)	C26—C25—H25	120.5
C2—C1—Co1	69.60 (16)	C24—C25—H25	120.5
C5—C1—H1	126.0	C27—C26—C25	120.4 (4)
C2—C1—H1	126.0	C27—C26—H26	119.8
Co1—C1—H1	126.0	C25—C26—H26	119.8
C1—C2—C3	108.3 (3)	C26—C27—C28	121.1 (4)
C1—C2—Co1	69.73 (16)	C26—C27—H27	119.4
C3—C2—Co1	69.94 (15)	C28—C27—H27	119.4
C1—C2—H2	125.9	C23—C28—C27	119.8 (3)
C3—C2—H2	125.9	C23—C28—H28	120.1
Co1—C2—H2	125.9	C27—C28—H28	120.1
C2—C3—C4	106.6 (2)	C34—C29—C30	118.5 (3)
C2—C3—P1	130.3 (2)	C34—C29—P2	123.7 (2)
C4—C3—P1	122.9 (2)	C30—C29—P2	117.8 (2)
C2—C3—Co1	69.18 (14)	C29—C30—C31	120.3 (3)
C4—C3—Co1	69.09 (15)	C29—C30—H30	119.8
P1—C3—Co1	123.76 (14)	C31—C30—H30	119.8
C5—C4—C3	108.1 (3)	C32—C31—C30	120.0 (3)
C5—C4—Co1	69.83 (16)	C32—C31—H31	120.0
C3—C4—Co1	69.95 (15)	C30—C31—H31	120.0
C5—C4—H4	126.0	C31—C32—C33	120.5 (3)
C3—C4—H4	126.0	C31—C32—H32	119.7
Co1—C4—H4	126.0	C33—C32—H32	119.7
C1—C5—C4	109.1 (3)	C32—C33—C34	119.7 (3)
C1—C5—Co1	70.10 (18)	C32—C33—H33	120.1
C4—C5—Co1	69.80 (16)	C34—C33—H33	120.1
C1—C5—H5	125.5	C29—C34—C33	120.9 (3)
C4—C5—H5	125.5	C29—C34—H34	119.5
Co1—C5—H5	125.5	C33—C34—H34	119.5
C11—C6—C7	118.7 (3)	F6—P3—F2	89.53 (12)
C11—C6—P1	118.7 (2)	F6—P3—F3	89.94 (12)
C7—C6—P1	122.3 (2)	F2—P3—F3	90.45 (11)
C8—C7—C6	120.4 (3)	F6—P3—F5	179.31 (13)
C8—C7—H7	119.8	F2—P3—F5	90.95 (12)
C6—C7—H7	119.8	F3—P3—F5	90.55 (12)
C9—C8—C7	120.5 (4)	F6—P3—F1	90.61 (12)
C9—C8—H8	119.8	F2—P3—F1	179.85 (13)
C7—C8—H8	119.8	F3—P3—F1	89.61 (11)
C8—C9—C10	120.3 (4)	F5—P3—F1	88.91 (12)
C8—C9—H9	119.8	F6—P3—F4	89.99 (11)
C10—C9—H9	119.8	F2—P3—F4	89.94 (11)
C9—C10—C11	119.7 (4)	F3—P3—F4	179.60 (12)
C9—C10—H10	120.1	F5—P3—F4	89.51 (11)
C11—C10—H10	120.1	F1—P3—F4	90.00 (11)
			
C18—Co1—C1—C5	141.23 (17)	C5—Co1—C18—C19	179.5 (2)
C19—Co1—C1—C5	178.85 (17)	C21—Co1—C18—C19	−80.75 (19)
C4—Co1—C1—C5	−37.11 (16)	C20—Co1—C18—C19	−36.95 (17)
C21—Co1—C1—C5	59.6 (3)	C2—Co1—C18—C19	98.2 (2)
C2—Co1—C1—C5	−119.1 (2)	C1—Co1—C18—C19	142.20 (19)
C3—Co1—C1—C5	−81.34 (18)	C3—Co1—C18—C19	59.9 (3)
C22—Co1—C1—C5	96.96 (18)	C22—Co1—C18—C19	−118.9 (3)
C18—Co1—C1—C2	−99.67 (18)	C19—Co1—C18—C22	118.9 (3)
C19—Co1—C1—C2	−62.1 (2)	C5—Co1—C18—C22	−61.5 (3)
C4—Co1—C1—C2	81.98 (18)	C21—Co1—C18—C22	38.18 (16)
C5—Co1—C1—C2	119.1 (2)	C20—Co1—C18—C22	81.98 (18)
C21—Co1—C1—C2	178.66 (18)	C2—Co1—C18—C22	−142.89 (16)
C3—Co1—C1—C2	37.76 (16)	C1—Co1—C18—C22	−98.87 (18)
C22—Co1—C1—C2	−143.94 (17)	C3—Co1—C18—C22	178.88 (16)
C5—C1—C2—C3	0.1 (3)	C22—C18—C19—C20	−1.1 (3)
Co1—C1—C2—C3	−59.54 (19)	Co1—C18—C19—C20	59.2 (2)
C5—C1—C2—Co1	59.6 (2)	C22—C18—C19—Co1	−60.33 (19)
C18—Co1—C2—C1	97.57 (19)	C18—Co1—C19—C20	−120.1 (2)
C19—Co1—C2—C1	141.53 (18)	C4—Co1—C19—C20	58.3 (3)
C4—Co1—C2—C1	−80.99 (18)	C5—Co1—C19—C20	−137 (7)
C5—Co1—C2—C1	−37.33 (17)	C21—Co1—C19—C20	−37.99 (16)
C20—Co1—C2—C1	179.7 (2)	C2—Co1—C19—C20	140.69 (17)
C3—Co1—C2—C1	−119.4 (2)	C1—Co1—C19—C20	179.09 (17)
C22—Co1—C2—C1	59.1 (3)	C3—Co1—C19—C20	96.38 (18)
C18—Co1—C2—C3	−143.05 (16)	C22—Co1—C19—C20	−82.02 (18)
C19—Co1—C2—C3	−99.08 (18)	C4—Co1—C19—C18	178.42 (18)
C4—Co1—C2—C3	38.40 (16)	C21—Co1—C19—C18	82.14 (18)
C5—Co1—C2—C3	82.05 (17)	C20—Co1—C19—C18	120.1 (2)
C20—Co1—C2—C3	−60.9 (2)	C2—Co1—C19—C18	−99.19 (18)
C1—Co1—C2—C3	119.4 (2)	C1—Co1—C19—C18	−60.8 (2)
C22—Co1—C2—C3	178.52 (17)	C3—Co1—C19—C18	−143.50 (17)
C1—C2—C3—C4	0.2 (3)	C22—Co1—C19—C18	38.10 (16)
Co1—C2—C3—C4	−59.20 (18)	C18—C19—C20—C21	0.8 (3)
C1—C2—C3—P1	176.6 (2)	Co1—C19—C20—C21	59.6 (2)
Co1—C2—C3—P1	117.2 (2)	C18—C19—C20—Co1	−58.9 (2)
C1—C2—C3—Co1	59.40 (19)	C18—Co1—C20—C19	37.24 (16)
O1—P1—C3—C2	−106.0 (3)	C4—Co1—C20—C19	−144.41 (17)
C12—P1—C3—C2	129.7 (3)	C5—Co1—C20—C19	178.86 (17)
C6—P1—C3—C2	19.4 (3)	C21—Co1—C20—C19	118.8 (2)
O1—P1—C3—C4	69.9 (3)	C2—Co1—C20—C19	−62.5 (2)
C12—P1—C3—C4	−54.4 (2)	C3—Co1—C20—C19	−100.27 (18)
C6—P1—C3—C4	−164.8 (2)	C22—Co1—C20—C19	81.43 (18)
O1—P1—C3—Co1	−15.6 (2)	C18—Co1—C20—C21	−81.55 (18)
C12—P1—C3—Co1	−139.92 (16)	C19—Co1—C20—C21	−118.8 (2)
C6—P1—C3—Co1	109.74 (17)	C4—Co1—C20—C21	96.80 (19)
C18—Co1—C3—C2	60.9 (2)	C5—Co1—C20—C21	60.1 (2)
C19—Co1—C3—C2	98.39 (19)	C2—Co1—C20—C21	178.67 (17)
C4—Co1—C3—C2	−118.2 (2)	C1—Co1—C20—C21	−168 (22)
C5—Co1—C3—C2	−80.70 (18)	C3—Co1—C20—C21	140.94 (17)
C21—Co1—C3—C2	179.47 (18)	C22—Co1—C20—C21	−37.36 (16)
C20—Co1—C3—C2	141.77 (18)	C19—C20—C21—C22	−0.1 (3)
C1—Co1—C3—C2	−37.57 (17)	Co1—C20—C21—C22	59.57 (19)
C18—Co1—C3—C4	179.16 (18)	C19—C20—C21—Co1	−59.6 (2)
C19—Co1—C3—C4	−143.38 (18)	C18—Co1—C21—C20	81.56 (18)
C5—Co1—C3—C4	37.53 (17)	C19—Co1—C21—C20	37.69 (17)
C21—Co1—C3—C4	−62.3 (2)	C4—Co1—C21—C20	−99.90 (19)
C20—Co1—C3—C4	−100.00 (19)	C5—Co1—C21—C20	−143.44 (18)
C2—Co1—C3—C4	118.2 (2)	C1—Co1—C21—C20	179.7 (2)
C1—Co1—C3—C4	80.66 (18)	C3—Co1—C21—C20	−61.5 (2)
C18—Co1—C3—P1	−64.4 (2)	C22—Co1—C21—C20	120.0 (2)
C19—Co1—C3—P1	−27.0 (2)	C18—Co1—C21—C22	−38.41 (16)
C4—Co1—C3—P1	116.4 (2)	C19—Co1—C21—C22	−82.28 (18)
C5—Co1—C3—P1	153.9 (2)	C4—Co1—C21—C22	140.14 (17)
C21—Co1—C3—P1	54.1 (2)	C5—Co1—C21—C22	96.59 (18)
C20—Co1—C3—P1	16.4 (2)	C20—Co1—C21—C22	−120.0 (2)
C2—Co1—C3—P1	−125.4 (3)	C1—Co1—C21—C22	59.8 (3)
C1—Co1—C3—P1	−162.9 (2)	C3—Co1—C21—C22	178.54 (16)
C2—C3—C4—C5	−0.4 (3)	C20—C21—C22—C18	−0.6 (3)
P1—C3—C4—C5	−177.13 (19)	Co1—C21—C22—C18	58.60 (19)
Co1—C3—C4—C5	−59.7 (2)	C20—C21—C22—P2	−177.0 (2)
C2—C3—C4—Co1	59.25 (18)	Co1—C21—C22—P2	−117.8 (2)
P1—C3—C4—Co1	−117.5 (2)	C20—C21—C22—Co1	−59.22 (19)
C19—Co1—C4—C5	179.6 (2)	C19—C18—C22—C21	1.1 (3)
C21—Co1—C4—C5	−100.36 (19)	Co1—C18—C22—C21	−58.90 (18)
C20—Co1—C4—C5	−144.07 (19)	C19—C18—C22—P2	177.6 (2)
C2—Co1—C4—C5	80.73 (19)	Co1—C18—C22—P2	117.7 (2)
C1—Co1—C4—C5	36.78 (18)	C19—C18—C22—Co1	59.97 (19)
C3—Co1—C4—C5	119.1 (3)	O1'—P2—C22—C21	102.3 (3)
C22—Co1—C4—C5	−62.1 (2)	C29—P2—C22—C21	−129.5 (3)
C18—Co1—C4—C3	−160 (4)	C23—P2—C22—C21	−21.3 (3)
C19—Co1—C4—C3	60.5 (3)	O1'—P2—C22—C18	−73.4 (3)
C5—Co1—C4—C3	−119.1 (3)	C29—P2—C22—C18	54.7 (3)
C21—Co1—C4—C3	140.58 (17)	C23—P2—C22—C18	162.9 (2)
C20—Co1—C4—C3	96.87 (18)	O1'—P2—C22—Co1	12.9 (3)
C2—Co1—C4—C3	−38.33 (16)	C29—P2—C22—Co1	141.02 (16)
C1—Co1—C4—C3	−82.28 (18)	C23—P2—C22—Co1	−110.76 (18)
C22—Co1—C4—C3	178.88 (16)	C18—Co1—C22—C21	118.3 (2)
C2—C1—C5—C4	−0.3 (3)	C19—Co1—C22—C21	80.58 (19)
Co1—C1—C5—C4	59.1 (2)	C4—Co1—C22—C21	−62.6 (2)
C2—C1—C5—Co1	−59.4 (2)	C5—Co1—C22—C21	−100.34 (19)
C3—C4—C5—C1	0.5 (3)	C20—Co1—C22—C21	37.21 (17)
Co1—C4—C5—C1	−59.3 (2)	C2—Co1—C22—C21	179.50 (18)
C3—C4—C5—Co1	59.73 (19)	C1—Co1—C22—C21	−143.43 (18)
C18—Co1—C5—C1	−61.3 (2)	C19—Co1—C22—C18	−37.72 (17)
C19—Co1—C5—C1	−45 (7)	C4—Co1—C22—C18	179.14 (18)
C4—Co1—C5—C1	120.2 (3)	C5—Co1—C22—C18	141.36 (18)
C21—Co1—C5—C1	−143.38 (17)	C21—Co1—C22—C18	−118.3 (2)
C20—Co1—C5—C1	179.09 (17)	C20—Co1—C22—C18	−81.09 (18)
C2—Co1—C5—C1	37.95 (16)	C2—Co1—C22—C18	61.2 (2)
C3—Co1—C5—C1	82.17 (18)	C1—Co1—C22—C18	98.27 (19)
C22—Co1—C5—C1	−99.43 (18)	C18—Co1—C22—P2	−118.4 (2)
C18—Co1—C5—C4	178.44 (18)	C19—Co1—C22—P2	−156.1 (2)
C21—Co1—C5—C4	96.39 (19)	C4—Co1—C22—P2	60.7 (2)
C20—Co1—C5—C4	58.8 (3)	C5—Co1—C22—P2	22.9 (2)
C2—Co1—C5—C4	−82.29 (18)	C21—Co1—C22—P2	123.3 (3)
C1—Co1—C5—C4	−120.2 (3)	C20—Co1—C22—P2	160.5 (2)
C3—Co1—C5—C4	−38.06 (17)	C2—Co1—C22—P2	−57.2 (3)
C22—Co1—C5—C4	140.33 (17)	C1—Co1—C22—P2	−20.2 (2)
O1—P1—C6—C11	53.1 (3)	O1'—P2—C23—C24	−42.9 (3)
C12—P1—C6—C11	178.9 (2)	C29—P2—C23—C24	−173.9 (2)
C3—P1—C6—C11	−73.0 (3)	C22—P2—C23—C24	81.9 (2)
O1—P1—C6—C7	−120.6 (3)	O1'—P2—C23—C28	137.0 (3)
C12—P1—C6—C7	5.3 (3)	C29—P2—C23—C28	6.0 (3)
C3—P1—C6—C7	113.3 (3)	C22—P2—C23—C28	−98.2 (3)
C11—C6—C7—C8	0.3 (5)	C28—C23—C24—C25	1.4 (5)
P1—C6—C7—C8	174.0 (3)	P2—C23—C24—C25	−178.7 (2)
C6—C7—C8—C9	−0.8 (6)	C23—C24—C25—C26	−2.1 (5)
C7—C8—C9—C10	0.8 (7)	C24—C25—C26—C27	1.1 (6)
C8—C9—C10—C11	−0.4 (6)	C25—C26—C27—C28	0.5 (6)
C7—C6—C11—C10	0.1 (5)	C24—C23—C28—C27	0.2 (5)
P1—C6—C11—C10	−173.8 (3)	P2—C23—C28—C27	−179.7 (2)
C9—C10—C11—C6	−0.1 (5)	C26—C27—C28—C23	−1.2 (5)
O1—P1—C12—C17	13.7 (3)	O1'—P2—C29—C34	158.1 (3)
C6—P1—C12—C17	−111.3 (2)	C23—P2—C29—C34	−73.4 (3)
C3—P1—C12—C17	137.8 (2)	C22—P2—C29—C34	31.6 (3)
O1—P1—C12—C13	−168.6 (2)	O1'—P2—C29—C30	−22.4 (4)
C6—P1—C12—C13	66.4 (2)	C23—P2—C29—C30	106.1 (2)
C3—P1—C12—C13	−44.4 (3)	C22—P2—C29—C30	−148.9 (2)
C17—C12—C13—C14	1.1 (4)	C34—C29—C30—C31	−0.5 (4)
P1—C12—C13—C14	−176.6 (2)	P2—C29—C30—C31	180.0 (2)
C12—C13—C14—C15	−0.5 (4)	C29—C30—C31—C32	0.0 (5)
C13—C14—C15—C16	0.1 (5)	C30—C31—C32—C33	0.3 (5)
C14—C15—C16—C17	−0.4 (5)	C31—C32—C33—C34	−0.2 (5)
C13—C12—C17—C16	−1.5 (4)	C30—C29—C34—C33	0.6 (4)
P1—C12—C17—C16	176.4 (2)	P2—C29—C34—C33	−179.9 (2)
C15—C16—C17—C12	1.1 (4)	C32—C33—C34—C29	−0.2 (5)
C4—Co1—C18—C19	−141 (4)		

### Hydrogen-bond geometry (Å, °)

**Table:**

*D*—H···*A*	*D*—H	H···*A*	*D*···*A*	*D*—H···*A*
C4—H4···F6^i^	0.98	2.49	3.450 (4)	167.
C18—H18···F5	0.98	2.43	3.327 (4)	152.

Symmetry codes: (i) *x*, *y*−1, *z*.
